# Synthesis and Propagation of Complement C3 by Microglia/Monocytes in the Aging Retina

**DOI:** 10.1371/journal.pone.0093343

**Published:** 2014-04-04

**Authors:** Matt Rutar, Krisztina Valter, Riccardo Natoli, Jan M. Provis

**Affiliations:** 1 The John Curtin School of Medical Research, The Australian National University (ANU), Canberra, Australian Capital Territory, Australia; 2 ANU Medical School, The Australian National University (ANU), Canberra, Australian Capital Territory, Australia; The University of Melbourne, Australia

## Abstract

**Introduction:**

Complement activation is thought to contribute to the pathogenesis of age-related macular degeneration (AMD), which may be mediated in part by para-inflammatory processes. We aimed to investigate the expression and localization of C3, a crucial component of the complement system, in the retina during the course of aging.

**Methods:**

SD rats were born and reared in low-light conditions, and euthanized at post-natal (P) days 100, 450, or 750. Expression of C3, IBA1, and Ccl- and Cxcl- chemokines was assessed by qPCR, and *in situ* hybridization. Thickness of the ONL was assessed in retinal sections as a measure of photoreceptor loss, and counts were made of C3-expressing monocytes.

**Results:**

C3 expression increased significantly at P750, and correlated with thinning of the ONL, at P750, and up-regulation of GFAP. *In situ* hybridization showed that C3 was expressed by microglia/monocytes, mainly from within the retinal vasculature, and occasionally the ONL. The number of C3-expressing microglia increased significantly by P750, and coincided spatiotemporally with thinning of the ONL, and up-regulation of Ccl- and Cxcl- chemokines.

**Conclusions:**

Our data suggest that recruited microglia/monocytes contribute to activation of complement in the aging retina, through local expression of C3 mRNA. C3 expression coincides with age-related thinning of the ONL at P750, although it is unclear whether the C3-expressing monocytes are a cause or consequence. These findings provide evidence of activation of complement during natural aging, and may have relevance to cellular events underling the pathogenesis of age-related retinal diseases.

## Introduction

Aging involves the gradual accumulation of changes associated with a heightened vulnerability to various diseases [1], [2]. Age-related macular degeneration (AMD) is a leading cause of blindness in people aged over 65 [3], with estimated worldwide prevalence of 30 to 50 million [4] which is expected to increase substantially in coming decades [5]. Involvement of inflammatory processes in the pathogenesis of AMD has been steadily accruing for many years [6], [7], [8], [9], [10], [11]. In recent times, identification of involvement of the complement system has firmly placed inflammation as a key factor influencing the onset and progression of AMD [12], [13].

The complement system is a component of the innate immune response providing a rapid host defence against a range of immunological challenges, and aiding in the maintenance of homeostasis (Reviewed in [14], [15]). Despite this beneficial function, a pathogenic role of the complement system in AMD has been revealed through a string of seminal gene association studies. These identified a significant association between the Y402H sequence variant in the regulatory gene complement factor H (CFH) with the incidence of AMD [16], [17], [18], [19], along with other susceptibility variants in complement pathway genes C2 [12], [20], CFB [12], [20], and the central component C3 [21], [22], [23], [24], [25] in later studies. However, a number of key aspects of the disease process remain unclear, including the cellular events that synthesise and promote complement activity in the retina (reviewed in [12]).

Recently, the concept of para-inflammation – a state of low-grade chronic inflammation – has gained considerable attention as a factor in the development of age-related diseases [26], [27]. Para-inflammation is thought to occur in tissues under noxious stress, including accumulation of free radicals during aging [1], and acts as an immunological mechanism to maintain tissue homeostasis [26]. However, this response is not stable and in aging persistent factors may promote a chronic dysregulation of para-inflammation, leading to pathology [26], [28]. As such, para-inflammation in the aging retina may play a role in the pathology of age-related retinopathies such as AMD [26], [28]. Significantly, expression of various complement components, including C3, has been shown to increase with age in mice [28], although the cellular mechanisms which mediate the elevation of complement in aging are presently unknown [28].

In previous studies we have used a rat model of retinal light damage to gain an understanding of how the retina responds to damage, specifically the expression and localisation of inflammatory mediators from complement and chemokine pathways [29], [30], [31], [32]. These investigations have shown – for the first time – that retinal monocytes/microglia synthesise C3 and deposit it in the outer retina during damage [30]. In the current study, we aim to describe the normal aging process in rats of the same strain as used in our light damage model, in order to clarify the factors which may promote chronic complement synthesis. Our results show that C3 mRNA is expressed by microglia/monocytes in the retina which gradually increase in number over the course of normal aging. This coincides spatiotemporally with thinning of the ONL at P750 and up-regulation of chemokines with age, although the casual role C3-expressing monocytes/microglia in these processes is unclear. These data inform our understanding of complement activity in the context of normal aging of the retina in our model, and may also provide insight into the complement-related processes in diseases such as AMD.

## Methods

### Rearing Conditions, Tissue Collection and Processing

The study was approved by the Animal Experimentation Ethics Committee (AEEC) of the Australian National University (R.BSB.05.10). All experiments conducted were in accordance with the ARVO Statement for the Use of Animals in Ophthalmic and Vision Research. Sprague-Dawley (SD) rats were born and reared in low-light conditions (5 lux) for 100, 450, or 750 post-natal days (P), with food and water provided *ad libitum*.

At the appropriate age, animals were euthanized by overdose of barbiturate administered by an intraperitoneal injection (60 mg/kg bodyweight, Valabarb; Virbac, Australia) which also included 2% lidocaine (Troy Laboratories, NSW, Australia) to minimize animal suffering. The left eye from each animal was marked at the superior surface for orientation then enucleated and processed for cryosectioning, while the retina from right eye was excised through a corneal incision and prepared for RNA extraction. Eyes collected for cryosectioning were quickly immersion-fixed in 4% paraformaldehyde in 0.1 M PBS (pH 7.3) for 3 hours at room temperature, then processed as described previously [31], and cryosectioned at 16 μm. Retinas for RNA extraction were immediately immersed in chilled RNAlater solution (Ambion, Austin, TX), then stored in accordance with the manufacturer’s instructions. The RNA samples were then extracted and analysed following a previously established methodology [29], [33].

### Quantitative Real Time Polymerase Chain Reaction (qPCR)

Quantitative PCR was used to measure expression of C3, 6 chemokines, and two house-keeping genes (Table 1). First-strand cDNA synthesis was performed as described previously [29]. Gene amplification was measured using commercially available TaqMan hydrolysis probes (Applied Biosystems, Foster City, CA), the details of which are provided in Table 1. The hydrolysis probes were applied in accordance with a previously established qPCR protocol [29]. The fold change was determined using the ΔΔC_q_ method where the expression of the target gene was normalised relative to the expression of two reference genes – glyceraldehyde-3-phosphate dehydrogenase (GAPDH), and Actin beta (Actb). Previous studies have shown that expression of GAPDH and Actb does not change with respect to age in either the retina or brain [34], [35]. Amplification specificity of each primer pair was assessed using gel electrophoresis.

**Table 1:** Taqman® probes used.

Gene Symbol	Gene Name	Catalogue	Entrez Gene ID
Actb	Actin, beta	Rn00667869_m1	NM_031144.2
C3	Complement component 3	Mm00437858_m1	NM_009778.2
Ccl2	Chemokine (C-C motif) ligand 2	Rn01456716_g1	NM_031530.1
Ccl3	Chemokine (C-C motif) ligand 3	Rn00564660_m1	NM_013025.2
Ccl4	Chemokine (C-C motif) ligand 4	Rn00587826_m1	NM_053858.1
Ccl7	Chemokine (C-C motif) ligand 7	Rn01467286_m1	NM_001007612.1
Cxcl10	Chemokine (C-X-C motif) ligand 10	Rn01413889_g1	NM_139089.1
Cxcl11	Chemokine (C-X-C motif) ligand 11	Rn00788262_g1	NM_182952.2
Gapdh	Glyceraldehyde-3-phosphate dehydrogenase	Rn99999916_s1	NM_017008.3
Gfap	Glial fibrillary acidic protein	Rn00566603_m1	NM_017009.2

### ONL Thickness Measurements

Thickness of the ONL was used to measure photoreceptor loss in each age group. ONL thickness was measured in 1 mm increments along the full-length of retinal cryosections cut in the para-saggital plane (superio-inferior), close to the vertical meridian. The DNA-specific dye bisbenzamide (Calbiochem, La Jolla, CA) was used to visualize the cellular layers. ONL thickness was calculated as the ratio of ONL thickness to the distance between the outer- and inner- limiting membranes (OLM-ILM), to take into account any obliquely cut sections or regions. The total ONL ratio from each retina is the average of 3 retina sections at comparable locations.

### *In situ* Hybridisation

To investigate localisation of C3 mRNA transcripts, a digoxygenin-conjugated riboprobe to C3 was generated for *in situ* hybridisation on cryosections of retinal tissue, as described in a previous study conducted by our group [30]. Synthesis of the C3 riboprobe and *In situ* hybridisation were performed in accordance with methodology described previously [36]; the C3 riboprobe was hybridised overnight at 57°C, and then washed in saline sodium citrate (pH 7.4) at 60°C. The bound probe was visualised with NBT/BCIP (Nitro blue tetrazolium/5-Bromo-4-chloro-3-indolyl phosphate), which was followed by a counterstain using standard immunohistochemical labelling for either IBA1 or isolectin B_4_ (described further in ‘immunohistochemistry’) as required.

### Immunohistochemistry

Cryosections from each treatment group were used for immunohistochemical analysis, using an antibody against IBA1 (1∶500, Cat# 019-19741; Wako, Osaka, Japan). Blood vessels were visualised using an isolectin B_4_ stain, conjugated to FITC (1∶100, Cat# L2895; Sigma-Aldrich, St. Louis, MO). Immunohistochemistry was performed using methodology previously described [29]. Immunofluorescence was viewed using a Zeiss laser scanning microscope, and acquired using PASCAL software (Zeiss, v4.0). Images were enhanced for publication using Adobe Photoshop software, which was standardised between images.

### Quantification of C3-expressing Cells

Counts of C3-expressing cells were performed on retinal cryosections stained for C3 using *in situ* hybridisation (as described above); identification of these C3-expressing cells as monocytes/microglia was confirmed in a previous investigation by our group [30]. Counts of C3-expressing cells were carried out along the full-length of retinal sections cut in the para-saggital plane (supero-inferior) close to the vertical meridian, in adjacent fields measuring 1 mm across. The counts of C3-expressing cells were grouped according to their localisation in either the retinal vasculature, ONL, or choroidal vasculature. The total number of C3-expressing cells from each retina is the average of 3 sections at comparable locations.

### Statistical Analysis

Statistical analysis was performed using the one-way ANOVA with Tukey’s multiple comparison post-test, or the unpaired Student’s t-test. For each analysis, differences with a P value <0.05 were considered statistically significant.

## Results

### Quantification of ONL Thickness and GFAP Expression with Age

**Figure 1:**
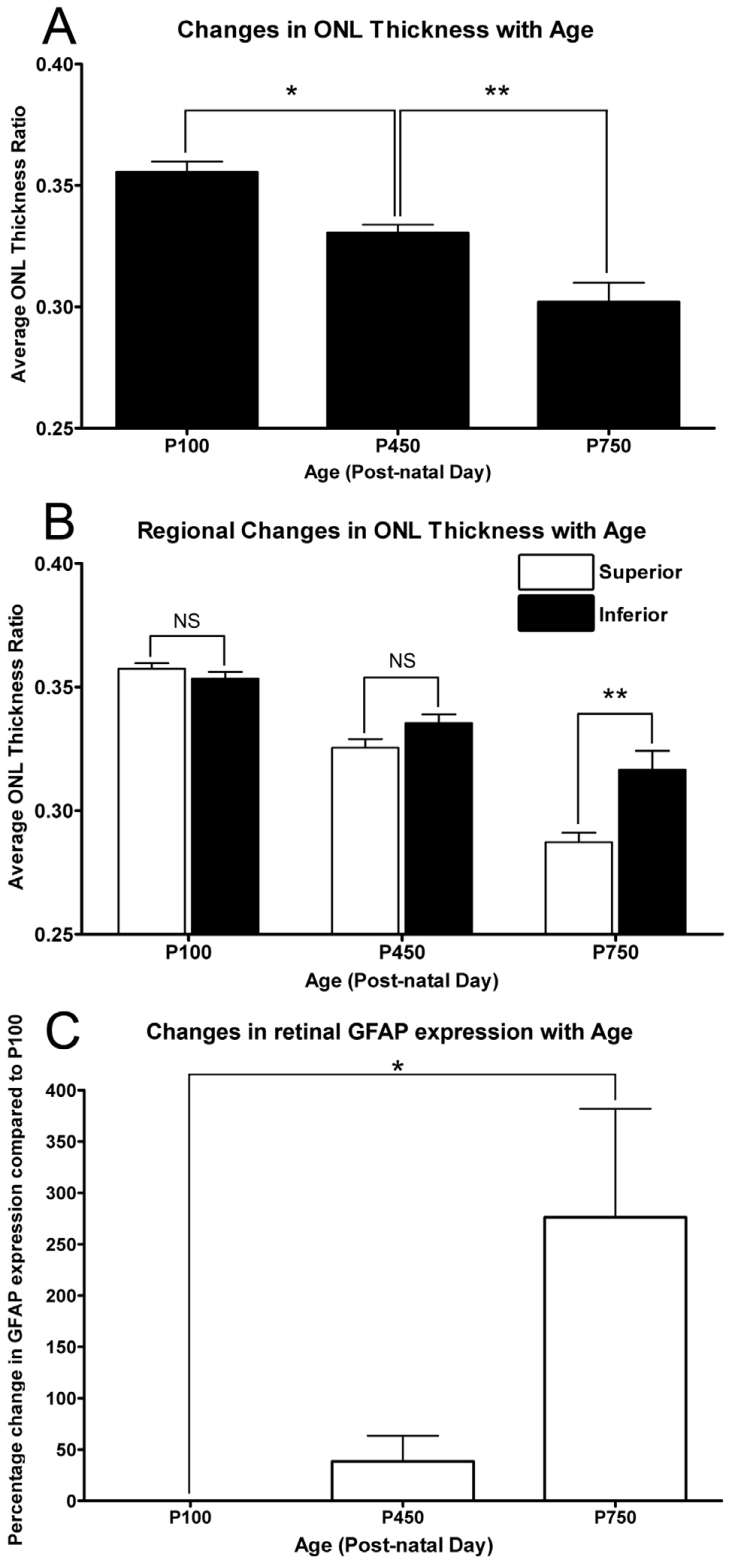
Quantification of ONL thickness and GFAP expression with respect to age. **A:** The thickness of the ONL decreased progressively throughout the aging time course, and was markedly reduced by P750 (P<0.05). **B:** Regional variation in ONL thickness was not observed at P100 and P450 age-groups across the vertical meridian (P>0.05). At P750, the greater thinning was observed in the superior retina than the inferior (<0.05). **C:** The expression of GFAP was found to increase at P450, however this was not significant compared to P100 (P>0.05). At P750 a 276% increase was observed in the expression of GFAP, compared to P100 (P<0.05). P100 n = 3, P450 n = 3, P750 n = 3; error bars represent SEM. ‘*’ denotes a significant change using ANOVA with Tukey’s post-test where P<0.05, ‘**’ denotes P<0.01.

The average thickness of the ONL gradually reduced throughout the aging timecourse (Figure 1A). ONL thickness decreased significantly by P450, compared to animals aged P100 (P<0.05). At P750, the ONL was significantly thinner than both P450 and P100 age-groups (P<0.05). Additionally, there was regional variation in the rate of ONL thinning, between superior and inferior retina, as a function of age (Figure 1B). At P450 superior retina shows a trend towards being thinner than inferior retina, but the difference is not significant (P>0.05). However, in P750 animals, the superior portion of the retina was significantly thinner than inferior retina (P<0.05).

We also detected modulation of the stress-marker GFAP over the aging timecourse (Figure 1C). Expression of GFAP increased significantly to 276% by P750 compared to P100 (P<0.05). Although the expression of GFAP at P450 also showed a tentative increase compared to P100, this proved to be highly inconsistent, and was not significant in relation to either P100 or P750 age groups.

### Differential Expression and Localisation of C3 in the Retina

**Figure 2:**
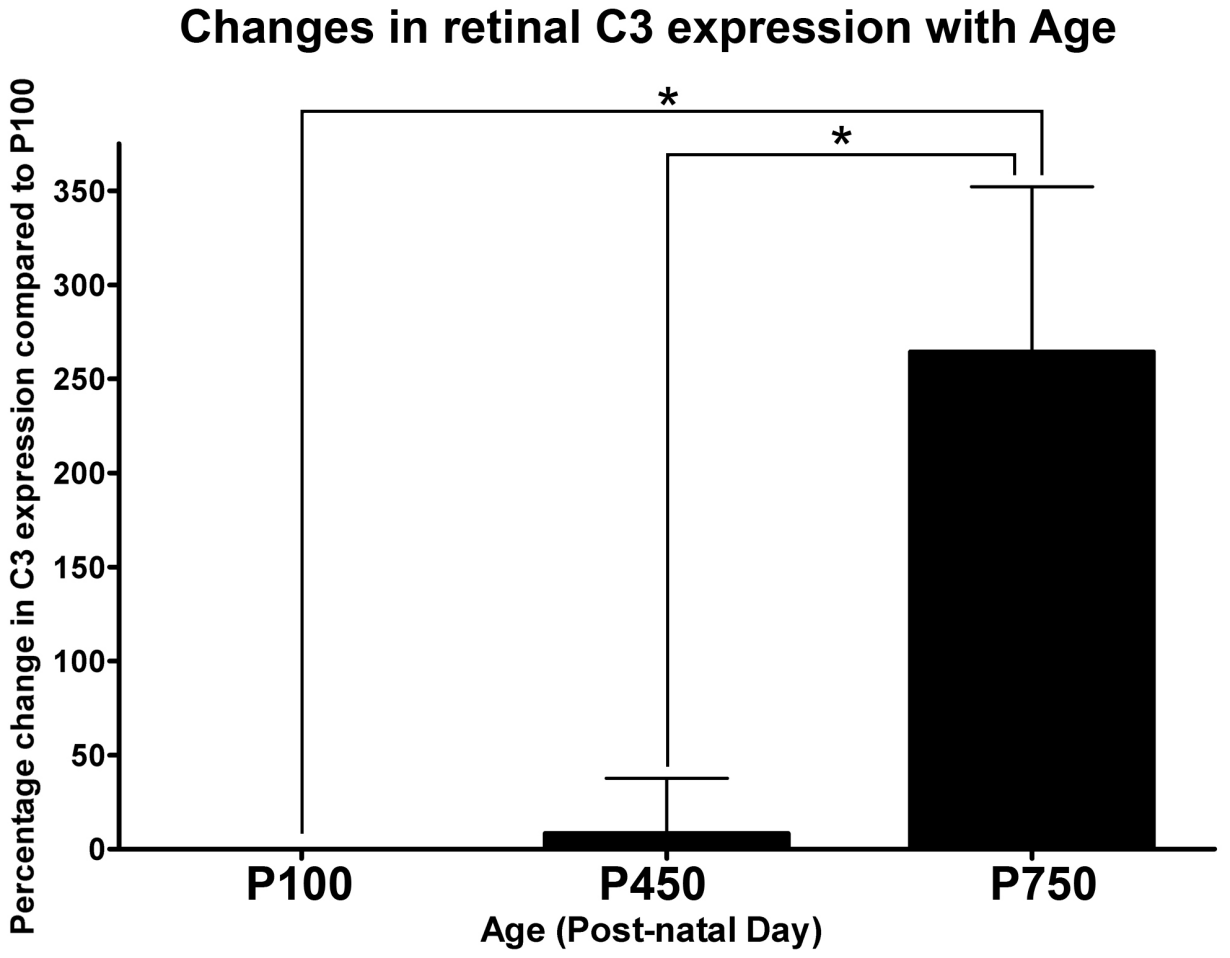
Expression of C3 in the neural retina by qPCR over the aging timecourse. No appreciable difference was observed in the expression of C3 between P100 and P450 age groups (P>0.05). By P750 there was a robust 264.3% increase in the expression of C3, compared to P100 and P450 (P<0.05). P100 n = 3, P450 n = 3, P750 n = 3; error bars represent SEM. ‘*’ denotes a significant change using ANOVA with Tukey’s post-test where P<0.05.

Using qPCR, the differential expression of C3 in the retina was assessed over the age timecourse (Figure 2). The expression of C3 did not change appreciably between animals aged P100, and those aged P450 (8.3%, P>0.05). At P750 however, the expression of C3 was significantly increased, reaching a differential expression of 264.3%, relative to animals aged P100 (P<0.05).

**Figure 3:**
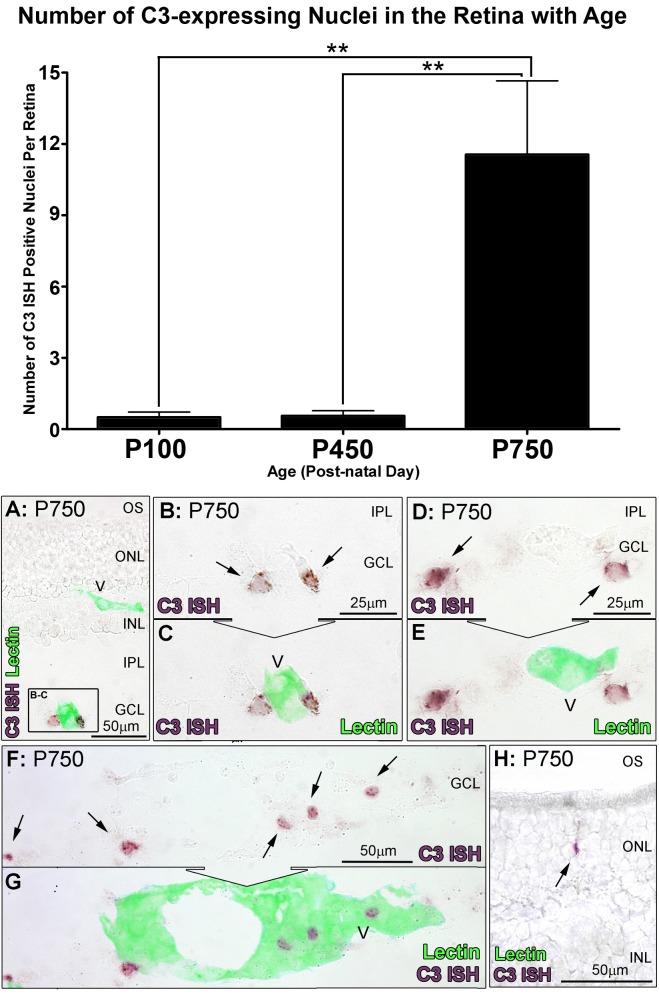
*In situ* hybridisation for C3 mRNA in the retina with respect to age. **A–F:** Representative images show *In situ* hybridisation for C3 mRNA (purple) and vessels (lectin staining, green) in the retina. In retinas from P750, expression of C3 was apparent in cells (A, C, E, arrows) closely associated with vessels from the retinal vasculature stained with lectin (B, D, E). **H:** Staining for C3 was also occasionally observed among cells situated in the ONL (arrow). **Histogram:** Quantification of C3-expressing cells per retina showed no significant change at P450 compared to P100 (0.6 per retina, P>0.05). At P750, C3-expressing cells increased significantly (P<0.05) to 11.6 per retina compared to both P100 and P450 groups. P100 n = 3, P450 n = 3, P750 n = 3; error bars represent SEM. ‘*’ denotes a significant change using ANOVA with Tukey’s post-test where P<0.05, ‘**’ denotes P<0.01. V, retinal blood vessel; INL, inner nuclear layer; IPL, inner plexiform layer; GCL, ganglion cell layer; ONL, outer nuclear layer; OS, outer segments.

**Figure 4:**
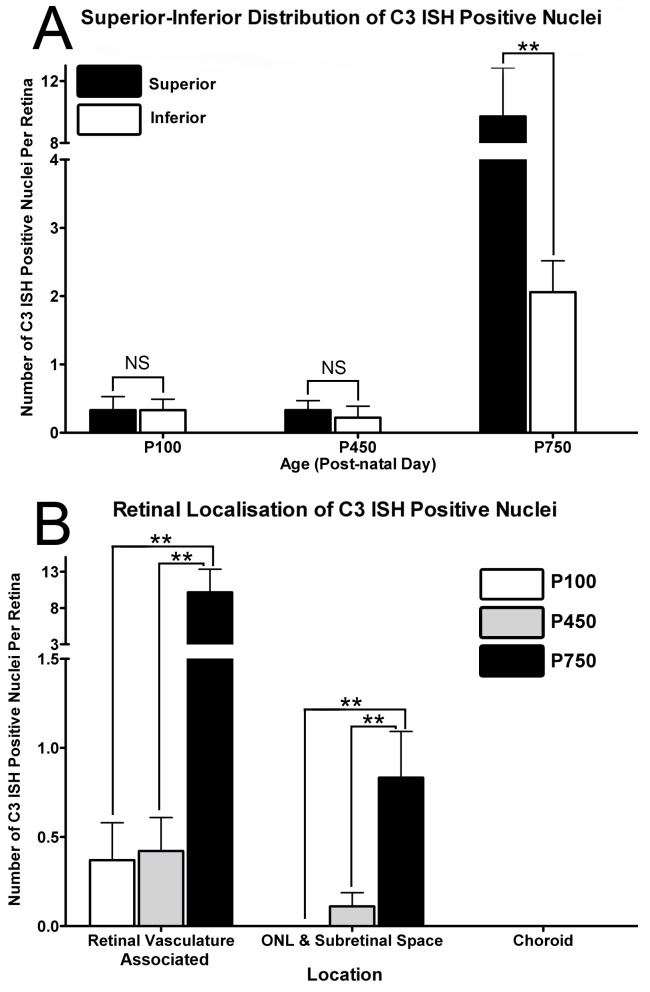
Distribution of *In situ* hybridisation for C3 mRNA in the over the aging timecourse. **A:** C3-expressing cells were evenly distributed across superior and inferior retina P100 and P450 animals (P>0.05). At P750, C3-expressing cells were more numerous in the superior portion of the retina than the inferior (9.7 and 2.1 respectively, P<0.05). **B:** Increases in C3-expressing cells were predominately associated with the retinal vasculature at P750 (P<0.05), with more modest increases apparent in the ONL and subretinal space over the same period (P<0.05). P100 n = 3, P450 n = 3, P750 n = 3; error bars represent SEM. ‘*’ denotes a significant change using ANOVA with Tukey’s post-test where P<0.05, ‘**’ denotes P<0.01.

**Figure 5:**
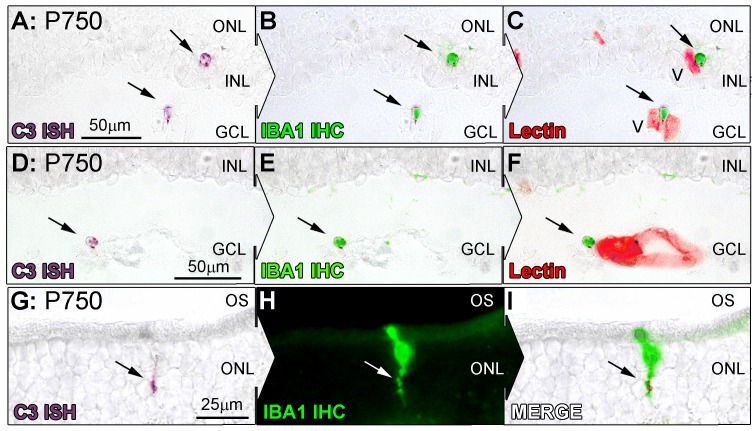
Co-immunolabeling of C3-expressing cells (purple) for IBA1 (green) at P750. **A–I:** Representative images at P750 show *In situ* hybridisation for C3 mRNA, IBA1 immunoreactivity, and lectin staining (red) for retinal vasculature in the retina. C3 staining (A, D, arrows) was found to correlate strongly with IBA1-immunoreactive microglia/monocytes (B, E, arrows), which were in close association with both superficial and deep retinal vasculature (C, F, arrows). C3-expressing cells in the ONL (G, arrow) also showed specificity for IBA1-immunreactive microglia at the ONL/OS margin (H,I, arrows). V, retinal blood vessel; INL, inner nuclear layer; GCL, ganglion cell layer; ONL, outer nuclear layer; OS, outer segments.

Localisation of C3 expression in the retina was assessed with *in situ* hybridisation over the aging timecourse (Figures 3, 4 and 5). *In situ* hybridisation was preferred over immunohistochemistry for this purpose, since the propensity of C3 protein to be deposited on activating surfaces may lead to false-positive observations. C3 mRNA was expressed in the aging retina by cells immunoreactive for the monocyte/microglia marker IBA1 (Figure 5A–I), consistent with our previous findings in a light-induced model of retinal degeneration [30]. Staining for C3 mRNA was evident within the cytoplasm of cells, which was often clustered in small process-like ramifications (Figure 5A–C, G–I; Arrows). Few C3-expressing cells were detected at P100, close to zero per retina (Figure 3, histogram); this was not significantly changed at P450 (0.6 per retina, P>0.05). However, by P750 the number of C3-expressing cells had significantly increased to 11.6 per retina, P<0.05 (Figure 3A–H, and histogram). The distribution of C3-expressing cells in superior and inferior retina is shown in Figure 4, histogram A. In P100 and P450 animals C3-expressing cells were evenly distributed across superior and inferior retina. At P750 however, C3-expressing cells were far more numerous in the superior portion of the retina than the inferior (9.7 and 2.1 respectively, P<0.05).

**Figure 6:**
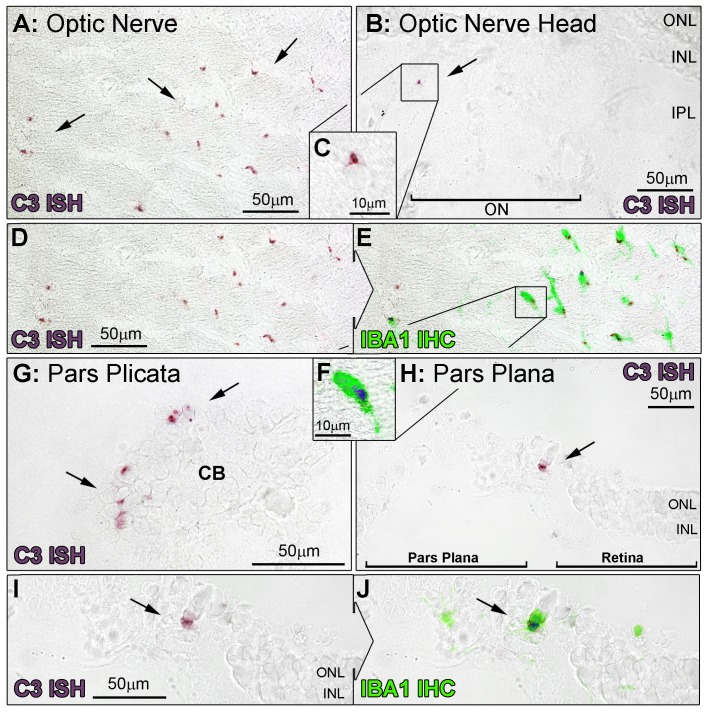
*In situ* hybridisation for C3-expressing cells in the optic nerve and ciliary body. Representative images demonstrate *In situ* hybridisation for C3 mRNA (purple) and IBA1-immunoreactive microglia/monocytes (green) at the optic nerve (A-F) and ciliary body (G-J), at P750. **A–F:** An abundance of C3-expressing cells were evident in section of the optic nerve tissue (A, arrows), and were immunoreactive for IBA1 (D–F). C3-expressing cells were also occasionally found emerging from the optic nerve head (B–C, arrow). **G–J:** At P750, C3-expressing cells were observed within the ciliary body, including the pars plicata (G, arrows) and the pars plana, particularly at the retinal margin (H, arrow). These cells were also immunoreactive for IBA1 (I–J). CB, ciliary body; V, retinal blood vessel; INL, inner nuclear layer; IPL, inner plexiform layer; ONL, outer nuclear layer.

The increased numbers of C3-expressing cells were mainly associated with the retinal vasculature (P<0.05) (Figure 4, histogram B), although there was also a modest increase in C3-expressing cells the ONL and subretinal space (P<0.05, Figure 4, histogram B). In general, C3-expressing cells were not observed in the choroid; rather, C3-expressing cells were mainly located in-and-around the retinal blood vessels at P750, in a pattern consistent with vascular cuffing (Figure 3A–C, D–E, F–G). C3-expressing cells were also detected at P750 in the optic nerve (Figure 6A, D–E) and at the optic nerve head (Figure 6B–C), in the ciliary body in association with the pars plicata (Figure 6G) and at the border of the pars plana and retina (Figure 6H, I–J).

### Expression of Ccl- and Cxcl- Chemokines in the Retina

**Figure 7:**
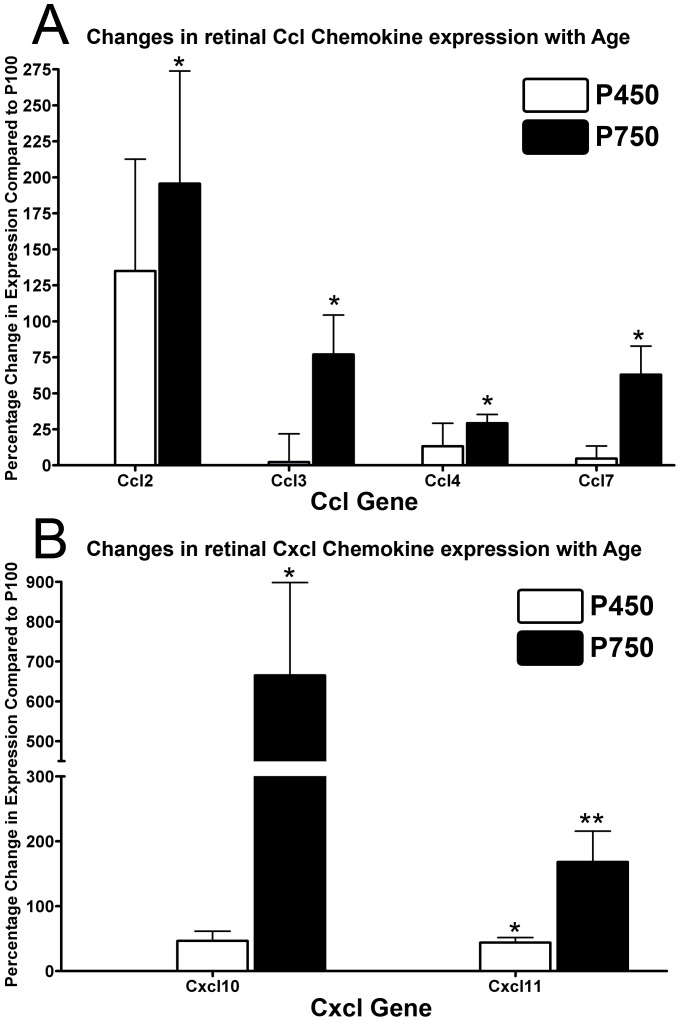
Expression of Ccl- (A) and Cxcl- (B) chemokines in the neural retina by qPCR over the aging timecourse. **A:** The expression of Ccl2, Ccl3, Ccl4, Ccl7 did not change appreciably in animals aged P450 (P>0.05), while a significant up-regulation was observed in P750 animals for all Ccl- genes (P<0.05), compared to P100; a large increase in Ccl2 expression was observed at P450, although this was not significant (P>0.05). **B:** At P450, there was a small increase in expression of Cxcl11 (P<0.05), but not Cxdc10 (P>0.05). By P750 there was a considerable increase in the expression of both Cxcl- genes compared to P100, particularly Cxcl10 (P<0.05). P100 n = 4, P450 n = 4, P750 n = 4; error bars represent SEM. ‘*’ denotes a significant change using ANOVA with Tukey’s post-test where P<0.05, ‘**’ denotes P<0.01.

In general, expression of Ccl2, Ccl3, Ccl4, Ccl7 (Figure 7A), and Cxcl10, Cxcl11 (Figure 7B) did not change significantly in P450 animals, compared those aged P100 (P>0.05), with the exception of Cxcl11 (43.7%, P<0.05). While there was some evidence of an increase in Ccl2 expression at P450 (134.9%), it was variable between animals and not statistically significant (P>0.05). By P750 however, significant increases expression of all Ccl- and Cxcl- chemokines examined was detected compared to P100 (P>0.05), the most highly upregulated being Ccl2 (195.6%) and Cxcl10 (665.0%).

## Discussion

The current study contributes to an understanding of complement activation in normal aging of the retina through several key aspects. Primarily, we show using in situ hybridisation, that IBA1-positive microglia/monocytes are a source of C3 mRNA in the aging retina, and are found associated primarily with the retinal vasculature as well as in the ONL at P750. We also find IBA1-positive C3-expressing cells beyond the retinal margins within the ciliary body, and in the optic nerve head in the aged retinas. In addition, we show that increased numbers of C3-expressing cells coincide spatiotemporally with increased expression of chemokines (Ccl- and Cxcl-), up-regulation of GFAP, and follow the initial thinning of the ONL. However, the role of C3-expressing monocytes/microglia in relation to these features of the aging process remains unclear.

### Complement Activity and Normal Aging of the Retina

Several investigations have previously described increased expression of complement components mRNAs – including C3– in the retinas of mice aged 20–24 months, compared to those aged 3 months [28], [37]. In this study we show, for the first time, that a source of C3 mRNA in the aging retina is from increased numbers of IBA1-immunoreactive microglia/monocytes located in the retinal vasculature and in the ONL. We find that these cells accumulate preferentially in superior retina at P750 at the site of maximal thinning of the ONL, and in spatiotemporal correlation with the location of the *area centralis* of the rat retina (see [31], [38]) – a homologue of the human macula. Similarly, age-related thinning of the ONL, and up-regulation of GFAP have been characterised in several human studies [39], [40], [41]. These findings from the normally aging rat retina are also consistent with our previous findings from younger rats of the same strain using a retinal light-damage model, in which C3-expressing monocytes appear in the superior retina at the locus of damage [30]. It should be noted however that the number of animals in the current study was relatively small, which is primarily attributed to the difficulties in rearing a large cohort to an advanced age such as P750. Nevertheless, the data are sufficiently robust and significant, and are in agreement with previous findings [28].

C3 is a key complement component that drives activation of all three pathways (reviewed in [15]). While activation of complement has beneficial properties including promoting the clearance of debris [14], [42], [43], [44], immune complexes [15], [45], [46], [47], and apoptotic cells [48], [49], [50], [51], it may also exacerbate degeneration if activated in an inappropriate manner. Such activation is implicated in various neurodegenerative disorders, as well as ischemia- and trauma-related injuries (reviewed in [44], [52], [53]). In the retina, recent experimental evidence indicates that copious synthesis of C3 is detrimental, in that a C3-expressing adenovirus transfected into mice retinas promotes degeneration of RPE and photoreceptors [54]. Such degeneration may be accelerated by the ablation of CFH, which spurs increased deposition of C3 in the outer retina of aged mice, as well as reduced retinal function [55]. Whether the accumulation of C3-expressing cells, as observed here, are a causative factor in the age-related thinning of the ONL, or a stress-related consequence of this phenomenon, is unclear. Nevertheless, increased synthesis of C3 by an accumulation of microglia/monocytes with age is likely to contribute to a propensity for increased complement activity in the retinal environment.

### Origins of C3-expressing Cells in the Aging Retina

The origin of the C3-expressing microglia/monocytes, and the avenue of their accumulation in the retina, remains uncertain. In this study we observe C3-expressing cells in and around the retinal vasculature, and at the both the ciliary margin and in the optic nerve. In particular, this distribution is consistent with recruitment of non-intrinsic, bone-marrow (BM)-derived monocytes into the retina, in findings garnered from several studies. Chimeric mice stressed by either *N*-methyl-*N*-nitrosourea (MNU) injection, retinal detachment, or light damage, have observed that BM-derived cells migrate into the retina along the retinal vasculature via the ciliary margin and optic disc, and are recruited to the ONL and subretinal space at the locus of damage [56], [57]. This distribution is also consistent with our understanding of the development of microglial populations in the retina [58]. Taken together, the striking similarity of these findings do suggest that *at least some* C3-expressing cells may be recruited BM-derived cells. However, we cannot discount the possibility that the C3-expressing cells include resident microglia, in which C3-expression has been induced by a parainflammatory environment. Further studies are needed to confirm these observations, using chimeras, parabiosis, or methods of monocyte depletion.

It is possible that escalation of complement activation in the aging retina, as indicated by increased C3 expression in the current study, is driven by aberrant para-inflammatory processes stimulated by the aging retinal environment (reviewed in [26]). Oxidative stress is a commonly accepted consequence of aging, and increases in markers such as oxidative low density lipoprotein and dinitrophenylated proteins have been observed in the retinas of aged mice [26]. Moreover, several investigations have demonstrated that complement activation may be stimulated by oxidative by-products. Mice immunized with the oxidative damage by-product CEP develop retinal degeneration and show increased deposition of complement C3 in the outer retina [59], [60]. Studies have also shown that RPE cultures exposed to photo-oxidative stress or oxidized photoreceptor outer segments have reduced expression of the complement regulatory gene CFH [61], [62]. Activation and deposition of complement may also be stimulated in microglia by increases in age-related deposits such as lipofuscin (reviewed in [63]). Recent evidence *in vitro* suggests that accumulation of lipofuscin/A2E in microglial cells promotes activation of complement, by simultaneously reducing synthesis of the complement inhibitor CFH while increasing expression of CFB [64].

Increases in C3-expressing monocytes/microglia in the aging retina are also likely to be stimulated by the increased expression of chemokines in the aging retina. Our data show that expression of both Ccl- and Cxcl- chemokines is up-regulated by P750, and is correlated with increases in C3-expressing cells. Previous microarray analysis of aged retinas have also reported increased expression of the chemokines Ccl2, Ccl3, and Ccl12 in 20 month-old mice compared to those aged 3-months [28]. We have recently shown that in the light-induced model of retinal degeneration, suppression of Ccl2 expression with a targeted small-interfering RNA (siRNA) inhibits recruitment of IBA1-positive monocytes/microglia, and reduces photoreceptor death in the retina [32]. Moreover, ablation of Ccl2 impairs the recruitment of BM-derived microglia/monocytes in retinas of in chimeric mice, where subclinical damage and parainflammation are induced by γ-ray irradiation [65].

### Relevance to Human Aging and AMD

Advancing age is the most consistent risk factors for AMD (reviewed in [66]). The findings of current study, and others [26], suggest that advancing age and para-inflammation predisposes the retina towards a pro-inflammatory and pro-complement environment, which in turn contributes to the pathogenesis of AMD. The cellular events leading to propagation of complement in the retina are not well understood (reviewed in [12], [67]) although it is well established that polymorphisms in a range of complement-related genes are associated with risk of AMD (reviewed in [12]). Moreover, recent findings from a large-scale gene expression study show that human donor eyes with any form of AMD have increased levels of expression of complement genes, including C3 [68]. The present findings from aging rat retina are consistent with those analyses [62], in that both show increases in C3 expression in the neural retina, while the current investigation further identifies monocytes/microglia as a source of this expression in the aging retina.

It is well established that recruitment of microglia/monocytes to the macula is strongly associated with all forms of AMD pathology [10], [11], [69], [70], [71], [72], and studies in experimental neovascular ‘wet’ AMD show that inhibition of monocyte recruitment reduces in the size of the neovascular lesion, compared to controls [73], [74]. Recent studies utilising AMD donor tissue have demonstrated that monocyte-recruiting chemokines, including Ccl2 and Cxcl10, are up-regulated in all forms of the disease [68]. Moreover, evidence from Sennlaub and colleagues indicates that elevated levels of Ccl2 in atrophic AMD lesions are accompanied by an influx of Ccr2^+^ monocytes [75]. This association is further confirmed in animal models, where suppression of Ccl2 reduces the extent of cell death in both experimental neovascularisation [76] and light-induced degeneration [32], [75]. Our current findings, in conjunctions with others [28], also show similar up-regulation of Ccl- and Cxcl- chemokines during normal retinal aging.

### Conclusion

Our findings suggest that the expression of C3 increases in the aging rat retina, due to the local synthesis of C3 by monocytes/microglia, and further implicate complement in the progression of retinal degeneration. While low levels of complement activation in the retina may be beneficial to the maintenance of retinal homeostasis, in circumstances of chronic para-inflammation it seems that over-activation may contribute to chronic stress and degeneration. This interpretation is consistent with findings showing that polymorphism in the C3 regulator CFH is associated with increased risk of AMD. Synthesis of C3 by microglia/macrophages and their increasing presence in the degenerating retina implicates them in complement activation in retinal aging. Consequently, modulation of microglial activation may be useful strategy to control detrimental propagation of complement in the aging retina, and in turn retinal degenerations such as AMD.
